# Supplementary material for: Mechano-responsive hydrogen-bonding array of thermoplastic polyurethane elastomer captures both strength and self-healing
Source: Nat Commun. 2021 Jan 27;12:621. doi: 10.1038/s41467-021-20931-z (PMC7841158; doi:10.1038/s41467-021-20931-z)
Supplement: Supplementary file 1 — Supplementary information [file 41467_2021_20931_MOESM1_ESM.pdf]

Supplementary Information for

Mechano-responsive hydrogen-bonding array of thermoplastic polyurethane elastomer captures both strength and self-healing

Eom *et al.*

1. Supplementary Tables

Supplementary Table 1. Polymer information and polymerization results

Entry[a]	Content of chain-extender[b] (wt%)	M_n (g mol^{-1})[c]	M_w (g mol^{-1})[c]	PDI[c]
C-IP-SS	14.6	16,500	24,100	1.46
E-IP-SS	14.6	30,400	45,600	1.50
Es-MD	-	55,400	98,600	1.78

[a]C: Poly(hexamethylene carbonate) diol, E: Poly(tetramethylene ether)glycol, Es: polyester-type macrodiol, IP: isophorone diisocyanate, MD: 4,4'-methylenebis(phenyl isocyanate), SS: bis(4-hydroxyphenyl)disulfide. [b]Based on the weight percentages of chain-extender included in the total monomer weight. [c]Determined by THF-GPC using polystyrene standards (RI detector).

Supplementary Table 2. Information on tensile properties of TPU films and the degree of recovery after self-healing. The data of quintuplicate samples are expressed as mean ± the standard deviation.

Entry		C-IP-SS[a]	E-IP-SS[b]	Es-MD
Virgin sample	Young's modulus (MPa)	15.5 ± 0.8	1.45 ± 0.1	8.86 ± 0.4
	UTS (MPa)	42.9 ± 1.4	6.76 ± 0.4	35.8 ± 0.8
	Elongation at break (%)	480 ± 3	920 ± 43	880 ± 8
	Toughness (MJ m^{-3})	75.1 ± 2.3	26.9 ± 2.7	115 ± 2
Cut & healed sample	Young's modulus (MPa)	15.3 ± 0.4	1.47 ± 0.1	-[c]
	UTS (MPa)	33.1 ± 0.4	5.96 ± 0.1	-
	Recovery of UTS (%)	77.2	88.2	-
	Elongation at break (%)	400 ± 6	920 ± 11	-
	Recovery of elongation (%)	82.7	99.6	-
	Toughness (MJ m^{-3})	48.4 ± 0.6	20.6 ± 0.2	-
	Recovery of toughness (%)	64.4	76.6	-

[a]Self-healing for 48 h at 35 °C. [b]Self-healing for 2 h at 25 °C. Tensile data obtained from the previous report (*Adv. Mater.* **2018**, *30*, 1705145). [c]No mechanical recovery was observed.

Supplementary Table 3. Tensile and self-healing properties of elastomers (tensile strength over 10 MPa, and self-healing efficiency over 60%)

	Self-healing motif/type of polymer[a]	The highest UTS (MPa)	Self-healing temperature (°C)	Recovery time	UTS recovery (%)	Ref.
Elastomer	Disulfide/TPU	43	35	24 h (48 h)	65 (77)	This work
Elastomer	Anisyl group /polyolefin	17.7	25	120 h	71	1
Elastomer	M-L, HB/PU	14.8	Room temp.[b]	130 h	93	2
Elastomer	M-L/PEI	10.5	25	12 h	~100	3
Elastomer	Electrostatic, HB/dual cross-linked polymer	27.4	25 (humid environ.)[c]	48 h	94	4
Elastomer	Ditelluride, HB/PUU	21.7	Ambient (up to 40 °C) (visible light)[d]	10 min	85.6	5
Elastomer	Diselenide, HB/PUU	~15	Ambient (photo reactor)[e]	10 min	~95	6
Elastomer	Dioxetane, disulfide/TPU	~25	40 (UV light)[f]	0.5 h	100	7
Elastomer	HB/PUU	48.5	80	48 h	88	8
Elastomer	Disulfide/PU	26.3	70	0.5 h	~98	9
Elastomer	Disulfide/TPU	25	70	6 h	86	10
Elastomer	M-L/Polyolefin	24.8	80	12 h	79	11
Elastomer	Disulfide, HB/PUU	25	100	2 h	92	12
Elastomer	M-L/polyisoprene	21	80	24 h	74	13
Elastomer	Disulfide/PU	~20	90	24 h	94	14
Elastomer	D-A/polyacrylates	13	60	1 week	79	15
Elastomer	Oxime-carbamate, HB/poly(oxime-urethane)	13.5	100	2 h	~100	16
Elastomer	Boronic ester/ENR	~10	80	24	85	17
Plastic[g]	HB/poly(thiourea-ether)	45	21	30 sec	unknown	18
Plastic	vdW/polyacrylates	9.74	Ambient	86 h	70	19
Plastic	Ionic interaction, HB/PU	16.9	40	2 h	70	20

[a]TPU: Thermoplastic polyurethane. M-L: Metal-ligand. HB: Hydrogen bonding. PEI: polyethyleneimine. PUU: poly(urethane-urea). D-A: Diels-Alder. ENR: epoxidized natural rubber. vdW: van der Waals.

[b]The exact room temperature was not recorded. Thus, general 25 ℃ was recorded in the Ashby graph.

[c]Humid environment: Relative humidity of ~90%.

[d]The light intensity of approximately 200000 lx.

[e]A photoreactor (300 W, 400–800 nm) with a higher light intensity of approximately 200000 lx.

[f]A high-pressure mercury lamp (500 W) with 365 nm optical filter.

[g]Recoginized as plastic-type owing to an appearance of a yield point.

Supplementary Table 4. The transition strain (έ) and stress (σ_t) of C-IP-SS at different temperatures

Temperature (K)	Transition strain (έ)	Transition stress (σ_t)
243	1.40	57.46
253	1.56	48.12
263	1.79	27.41
273	1.89	8.40
283	2.17	2.57
293	2.38	1.40
303	2.38	1.00
308	2.38	0.65
313	2.38	0.47

Supplementary Table 5. Average score of local irritation reaction to subcutaneous implants from histological data (n = 4)

Cell type/ tissue response	Experimental (C-IP-SS)	Control	
		Negative (HDPE)	Positive (DMSO-TPU)
Inflammation polymorphonuclear cells[a]	0.75	0.29	3.00
Lymphocytes[a]	0.25	0.14	2.50
Plasma cells[a]	0.00	0.00	0.00
Macrophages[a]	0.00	0.00	0.25
Giant cells[a]	0.00	0.00	0.00
Necrosis[b]	0.00	0.00	2.25
A: subtotal inflammation score (×2)	2.00	0.86	16.00
Neovascularization[c]	0.00	0.57	2.25
Fibrosis[d]	1.00	1.29	2.75
Fatty infiltrate[e]	0.00	0.00	0.25
B: tissue response subtotal	1.00	1.86	5.25
Total (A+B)	3.00	2.72	21.25
Score (= experimental – negative control)[f]	**0.29**	-	18.53
Traumatic necrosis	0	0	0
Foreign debris	0	0	0
No. sites examined	8	8	8

[a] Mean histopathological score of reaction based on the number of cells per high-powered field at 400x: 0 = 0 cells; 1= 1-5 cells; 2 = 5-10 cells; 3 = heavy infiltrate; 4 = packed.

[b] 0 = None; 1 = minimal; 2= mild; 3 = moderate; 4 = severe.

[c] 0 = None; 1 = minimal capillary proliferation, focal, 1–3 buds; 2= groups of 4–7 capillaries with supporting fibroblastic structures; 3 = broad band of capillaries with supporting structures; 4 = extensive band of capillaries with supporting fibroblastic structures.

[d] 0 = None; 1 = narrow band; 2= moderately thick band; 3 = thick band; 4 = extensive band.

[e] 0 = None; 1 = minimal amount of fat associated with fibrosis; 2= several layers of fat and fibrosis; 3 = elongated and broad accumulation of fat cells about the implant site; 4 = extensive fat completely surrounding the implant.

[f] Non-irritant (0.0 up to 2.9) < slight (3.0 up to 8.9) < moderate (9.0 up to 15.0) < severe (more than 15.0).

The local irritation effects were evaluated by a comparison of the tissue response caused by the tested implant to that caused by the negative control. The stained tissue cross-sections were examined by a pathologist in a public contract clinical research organization using an optical microscopy at high-powered field of 400x, and the pathologist scored local irritation responses based on the number of cells and the tissue change. The all scores are average values (n = 4). The subtotal inflammation score was multiplied by 2 and added to the subtotal score of neovascularization, fibrosis, and fatty infiltrate. Then, the value was subtracted by the score of negative control. According to the final score, the sample can be considered as non-irritant (0.0 up to 2.9), slight (3.0 up to 8.9), moderate (9.0 up to 15.0), and severe (more than 15.0).

Supplementary Table 6. The frequency shift factors (α_T) employed in obtaining TTS master curves for **C-IP-SS** and **E-IP-SS**, respectively

Measurement temperature (°C)	α_T of C-IP-SS	α_T of E-IP-SS
25	Reference temperature	
35	6.20×10^{-2}	4.47×10^{-1}
45	7.67×10^{-3}	1.28×10^{-1}
55	1.80×10^{-3}	4.74×10^{-2}
65	4.00×10^{-4}	1.76×10^{-2}
75	1.13×10^{-4}	8.21×10^{-3}
85	3.30×10^{-5}	3.42×10^{-3}

2. Supplementary Figures

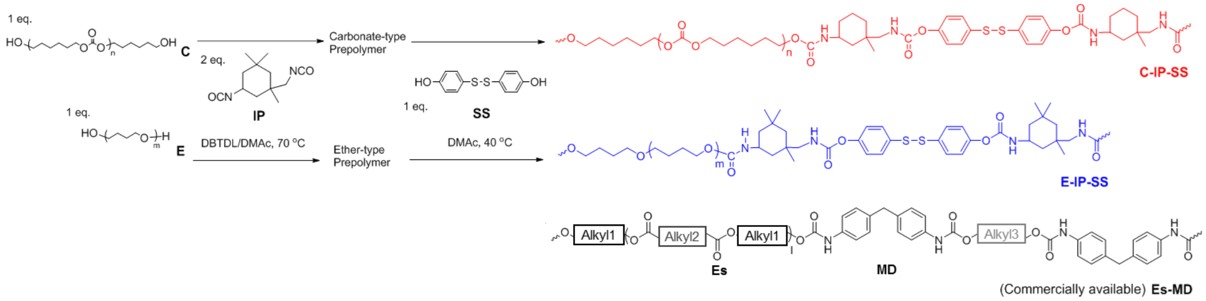

Supplementary Figure 1. Synthetic route to TPUs with different components. The TPUs are designated as X-Y-Z, where X, Y, and Z denote the abbreviation of the type of macrodiol, diisocyanate monomer, and chain extender, respectively (C-IP-SS, E-IP-SS, and Es-MD).

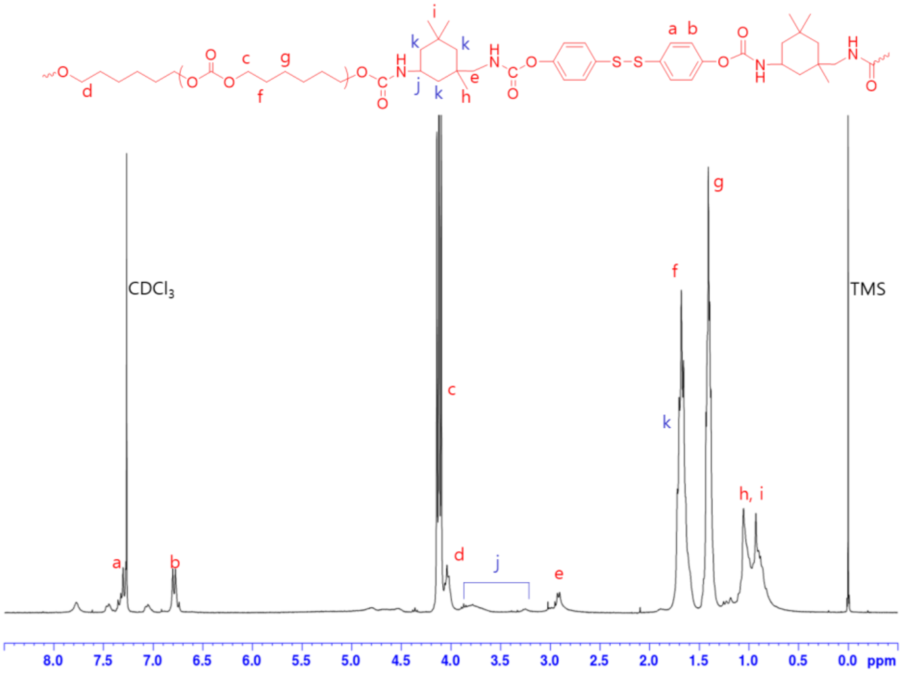

Supplementary Figure 2. ^1H NMR spectrum of C-IP-SS in $CDCl_3$ (300 MHz). In the ^1H NMR spectrum of C-IP-SS, the peaks of SS aromatic unit appeared at 7.4~6.6 ppm, the peaks of protons adjacent to carbonate group of poly(hexamethylene carbonate) diol (C) appeared at 4.2~4.0 ppm, and the peaks of IP alkyl unit appeared at 1.1~0.8 ppm. Integral ratio of each units was well-matched with feed molar ratio.

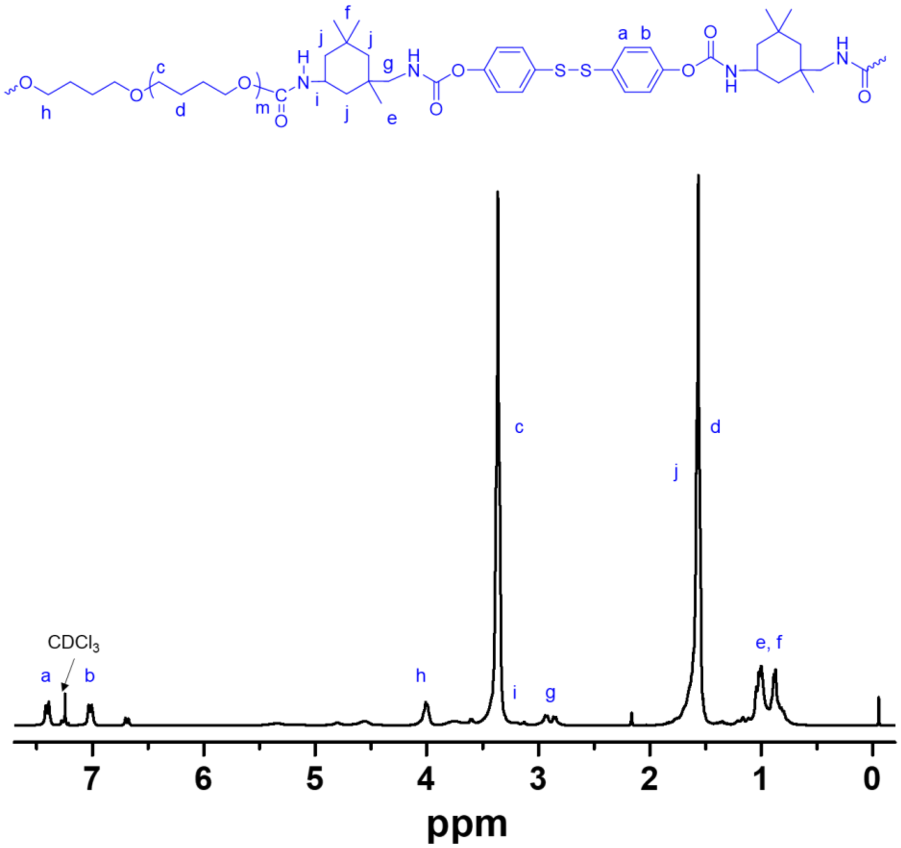

Supplementary Figure 3. ^{1}H NMR spectrum of E-IP-SS in $CDCl_3$ (300 MHz). In the ^{1}H NMR spectrum of E-IP-SS, the peaks of SS aromatic unit appeared at 7.4~6.6 ppm, the peaks of PTMEG (E) protons appeared at 3.4 and 1.6 ppm, and the peaks of IP alkyl unit appeared at 1.1~0.8 ppm. Integral ratio of each units was well-matched with feed molar ratio.

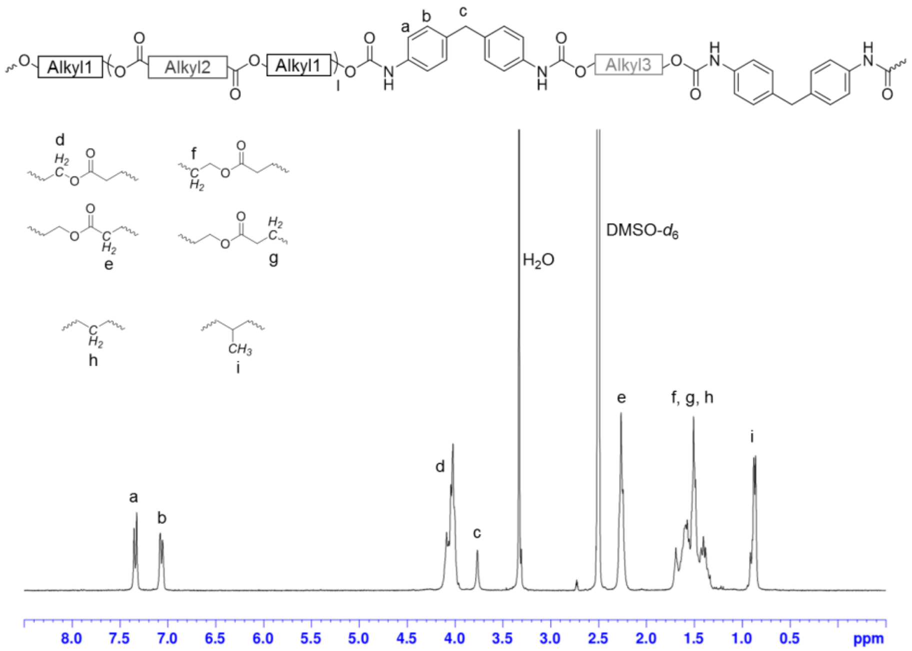

Supplementary Figure 4. ^{1}H NMR spectrum of Es-MD in DMSO-d_6 (300 MHz). In the ^{1}H NMR spectrum of Es-MD, the peaks of MD aromatic unit appeared at 7.4~7.0 and 3.8 ppm. The peaks of protons adjacent to ester C-O appeared at 4.2~4.0 ppm, and those adjacent to ester C=O appeared at 2.3-2.2 ppm, respectively, suggesting that soft segment is composed of polyester-type macrodiol (Es). Methylene protons appeared at 1.7~1.3 ppm and α-methyl protons (-CH_3) appeared at 1.0~0.8 ppm.

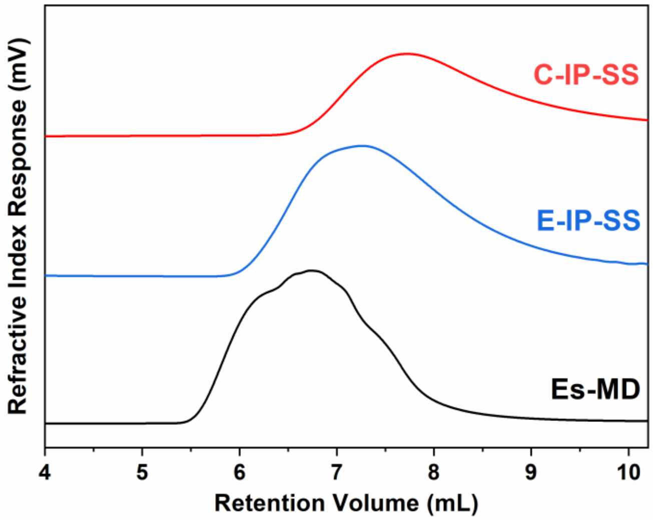

Supplementary Figure 5. THF-GPC profiles of TPUs.

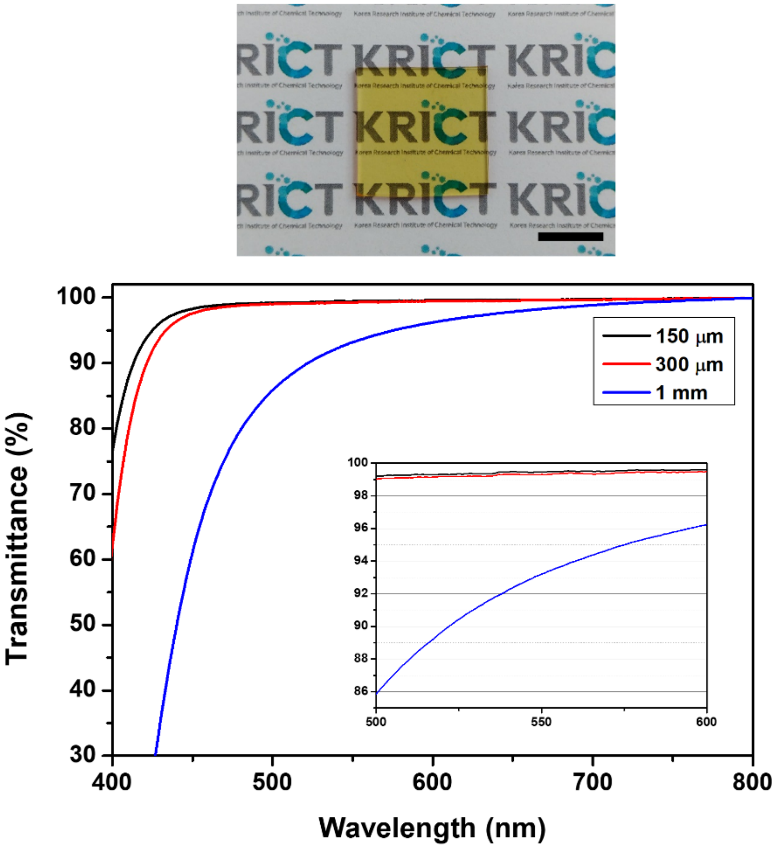

Supplementary Figure 6. Photograph of C-IP-SS films with a dimension of 20 mm × 20 mm × 1 mm (scale bar: 10 mm), and transmission spectra with various thicknesses.

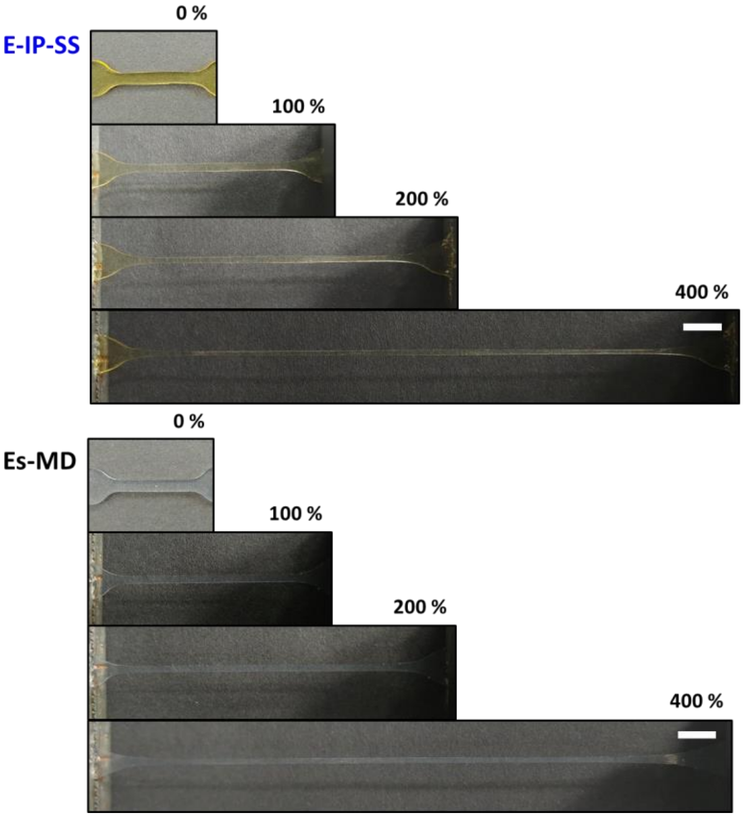

Supplementary Figure 7. Photographs of dumbbell-shaped films of E-IP-SS and Es-MD of various stretching ratio (scale bar: 6 mm).

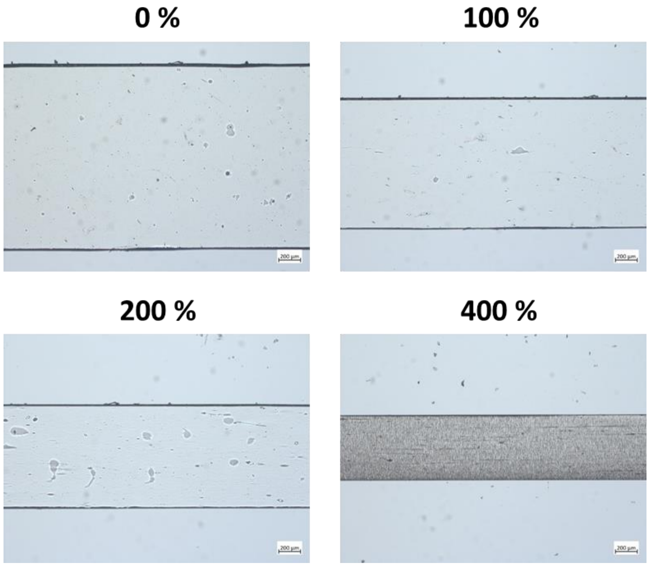

Supplementary Figure 8. OM images of dumbbell-shaped film of C-IP-SS (thickness: 300 µm) at different strains.

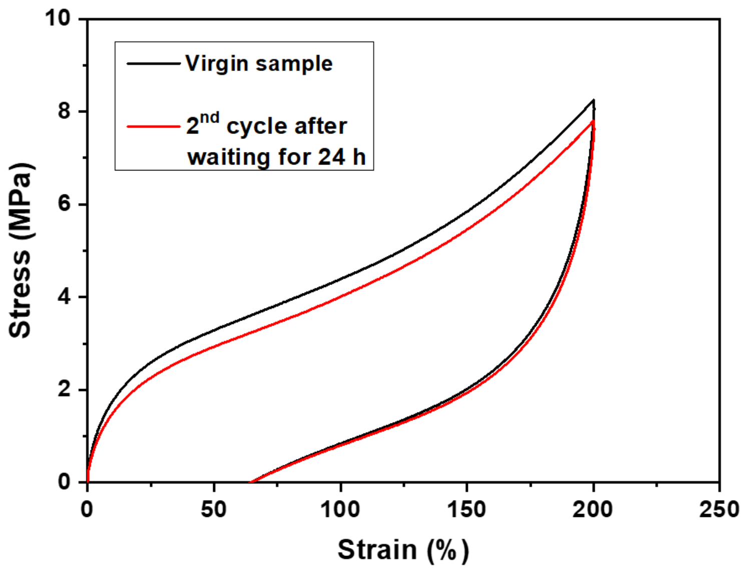

Supplementary Figure 9. Cyclic stress-strain curves of C-IP-SS with two cycles of 200% tensile strains. The second cycle was carried out after waiting for 24 h.

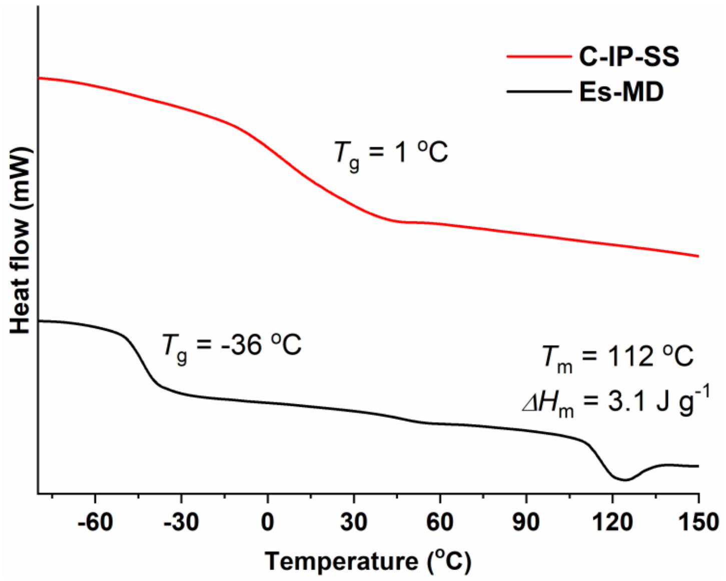

Supplementary Figure 10. Differential scanning calorimetry curves of C-IP-SS and Es-MD (first scan).

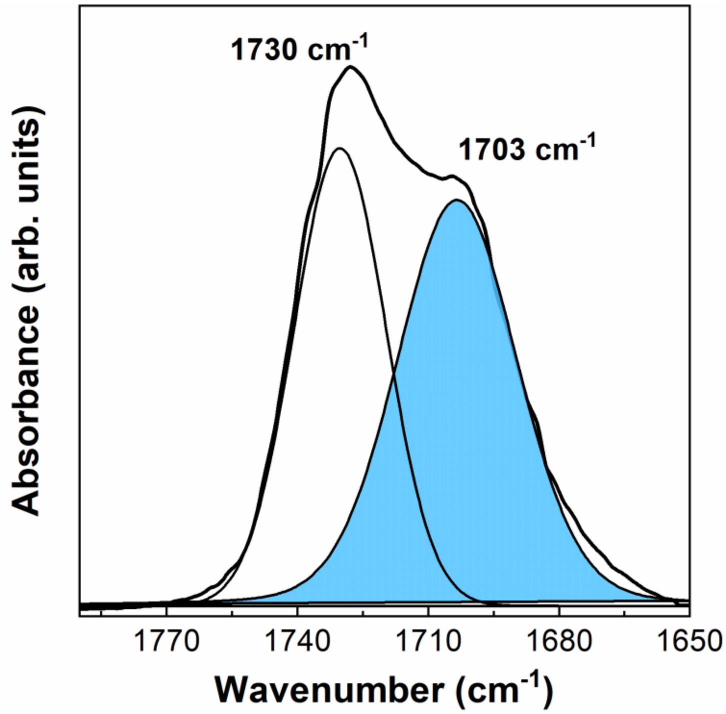

Supplementary Figure 11. The carbonyl region of FT-IR spectrum with peak deconvolution for Es-MD.

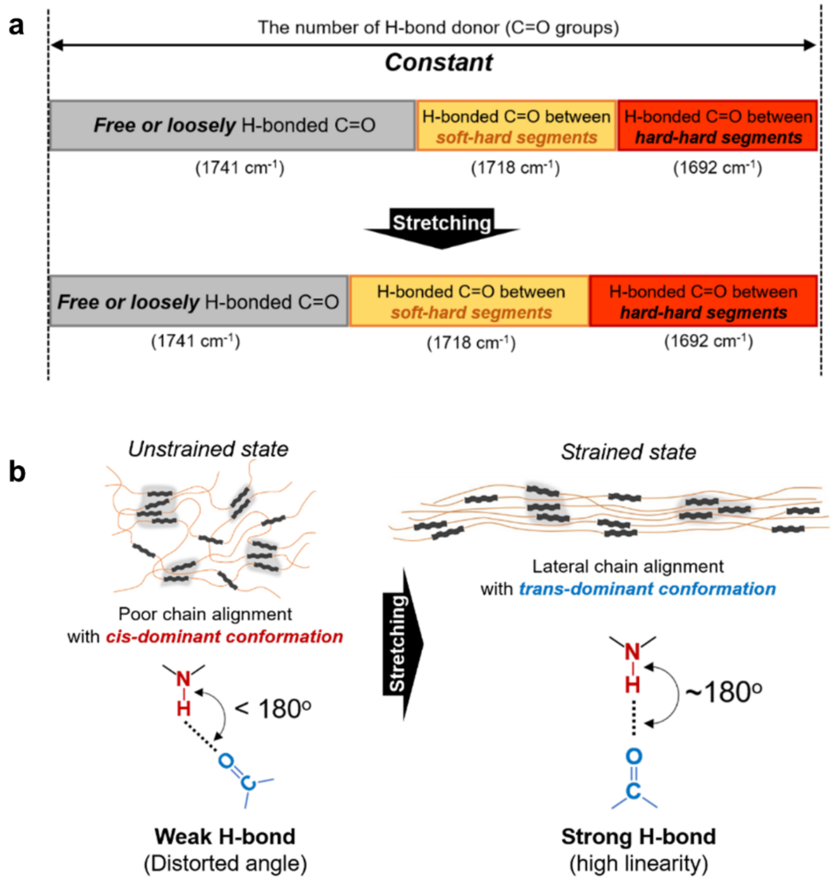

Supplementary Figure 12. (a) Stretching-dependent change of H-bond states and related FT-IR bands of C-IP-SS with a constant number of H-bond donors (carbonyl groups) and (b) the effect of chain conformational change upon straining on the linearity and strength of H-bond.

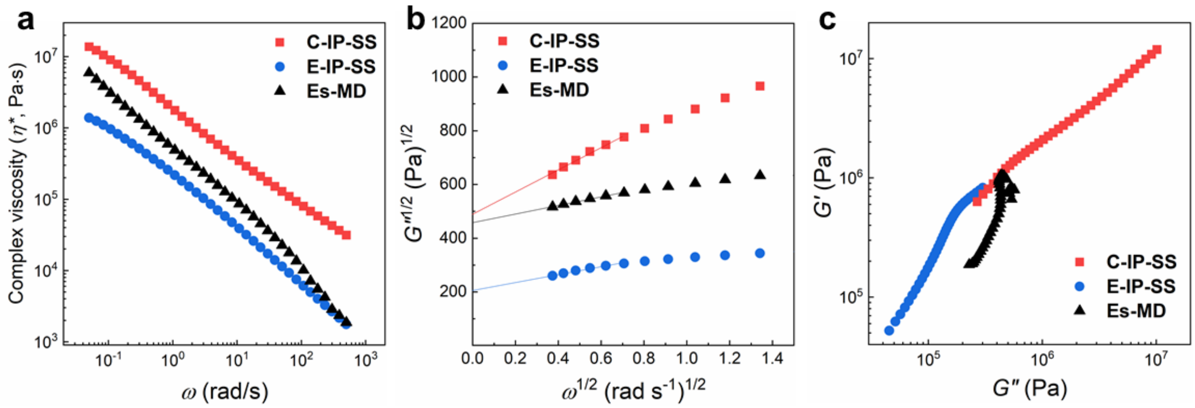

Supplementary Figure 13. Rheological properties of the three TPUs at 25 °C. (a) Complex viscosity versus angular frequency of 0.05−500 rad s^{-1}. (b) Casson plots to obtain the yield stress. The yield stress was calculated from the square value of the intercept of the plot. (c) The modified Cole−Cole plot.

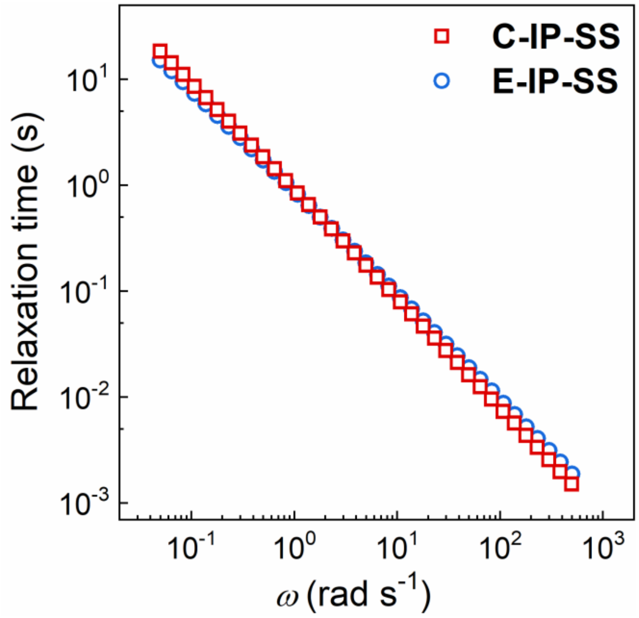

Supplementary Figure 14. Relaxation time curves of self-healable TPUs, C-IP-SS and E-IP-SS, over the frequency range of 0.05–500 rad s^{-1} at 25 °C.

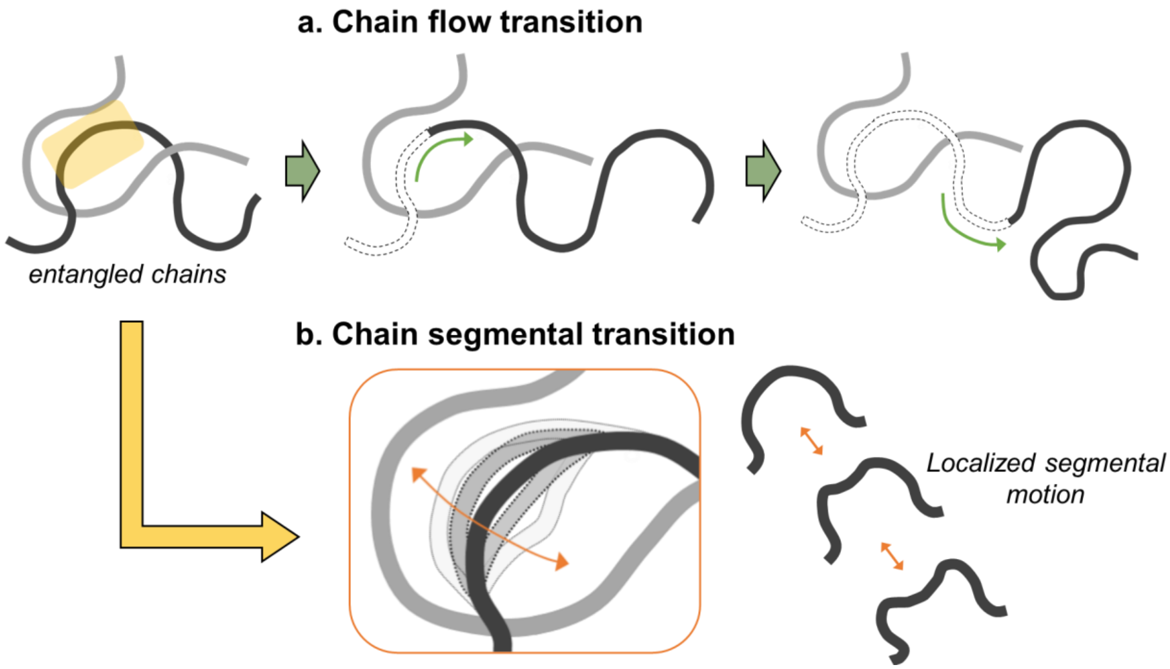

Supplementary Figure 15. Schematic illustration of the movement of (a) chain flow transition and (b) chain segmental transition.

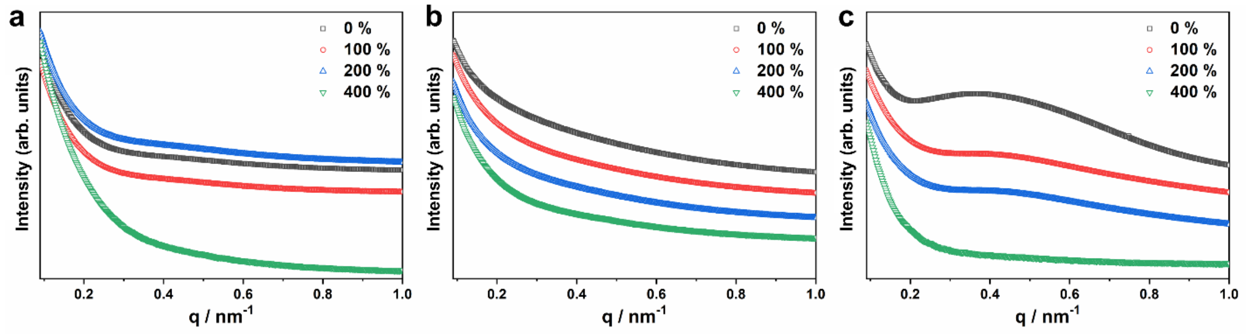

Supplementary Figure 16. The 1D SAXS patterns of (a) C-IP-SS, (b) E-IP-SS and (c) Es-MD at different degrees of stretching.

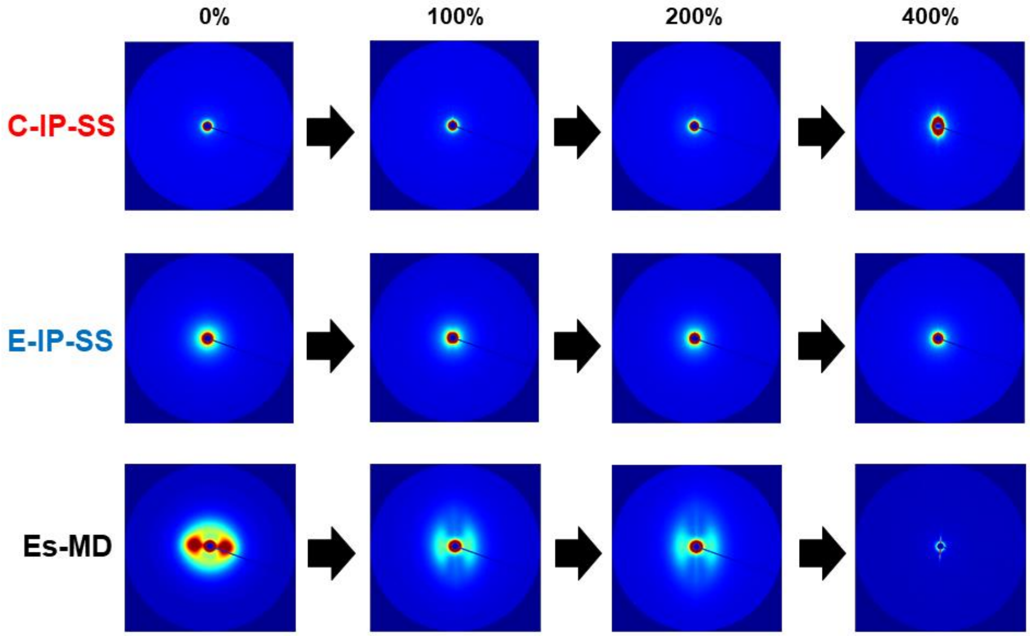

Supplementary Figure 17. The 2D SAXS patterns of the three types of TPUs at different degrees of stretching.

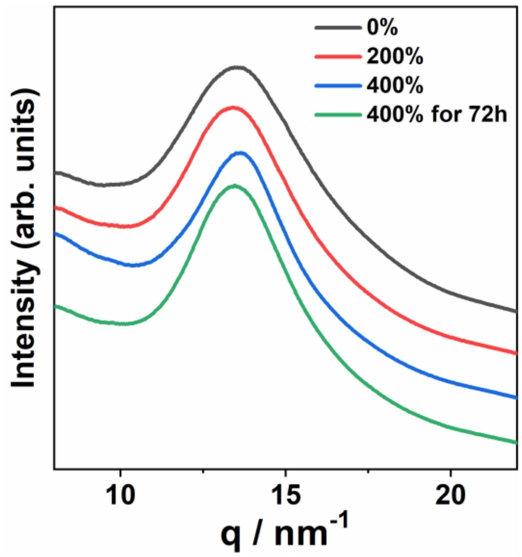

Supplementary Figure 18. The 1D WAXS patterns of Es-MD at different degrees of stretching.

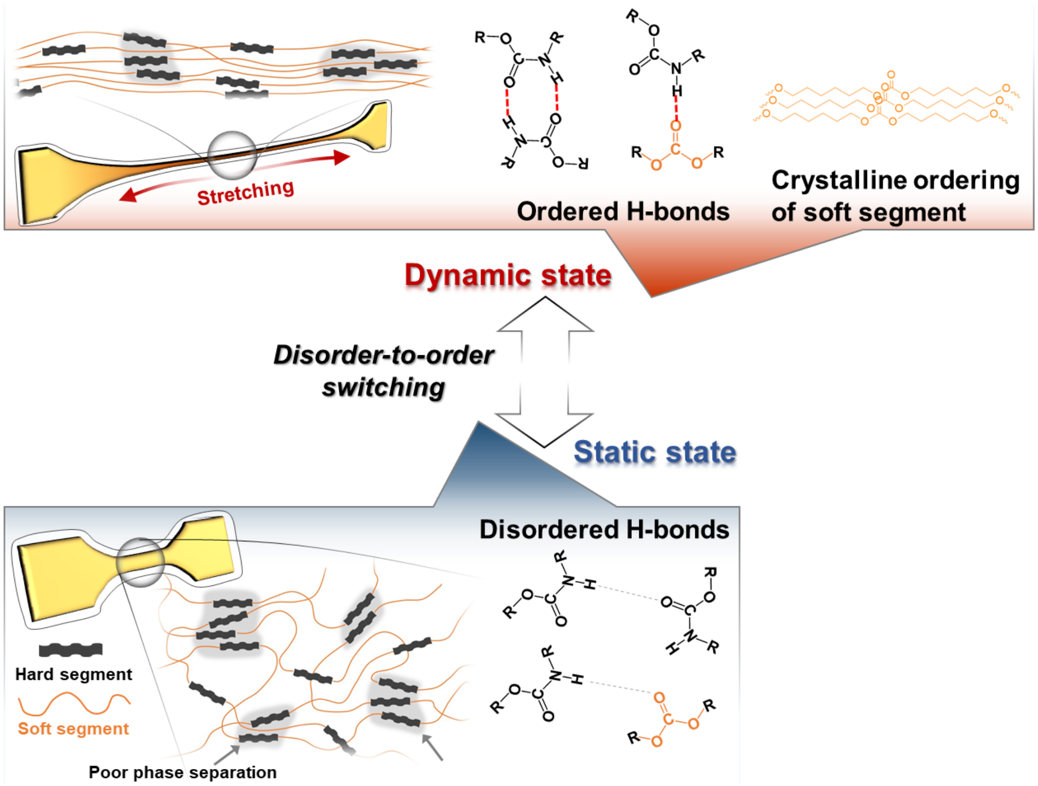

Supplementary Figure 19. Schematic illustration of mechano-responsive change in chain alignment and relevant H-bond array for C-IP-SS.

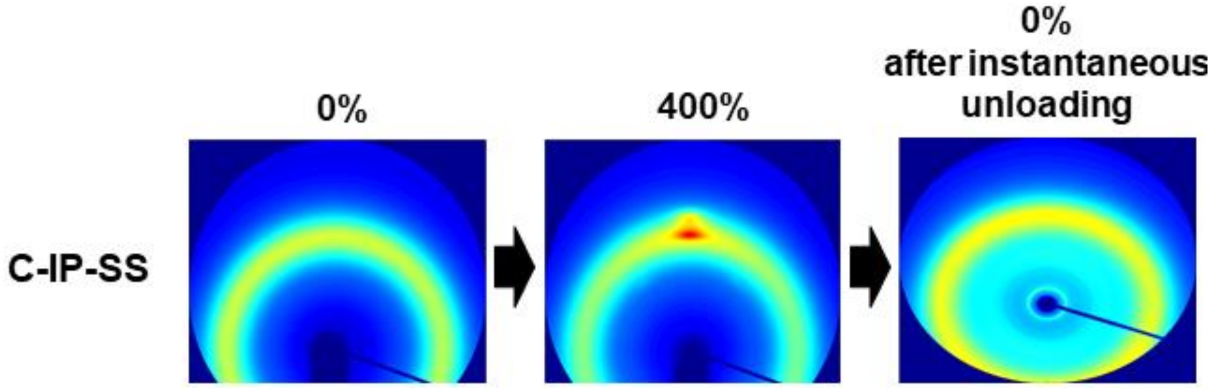

Supplementary Figure 20. The recovery of the 2D WAXS patterns for C-IP-SS after instantaneous release of the 400%-stretched specimen.

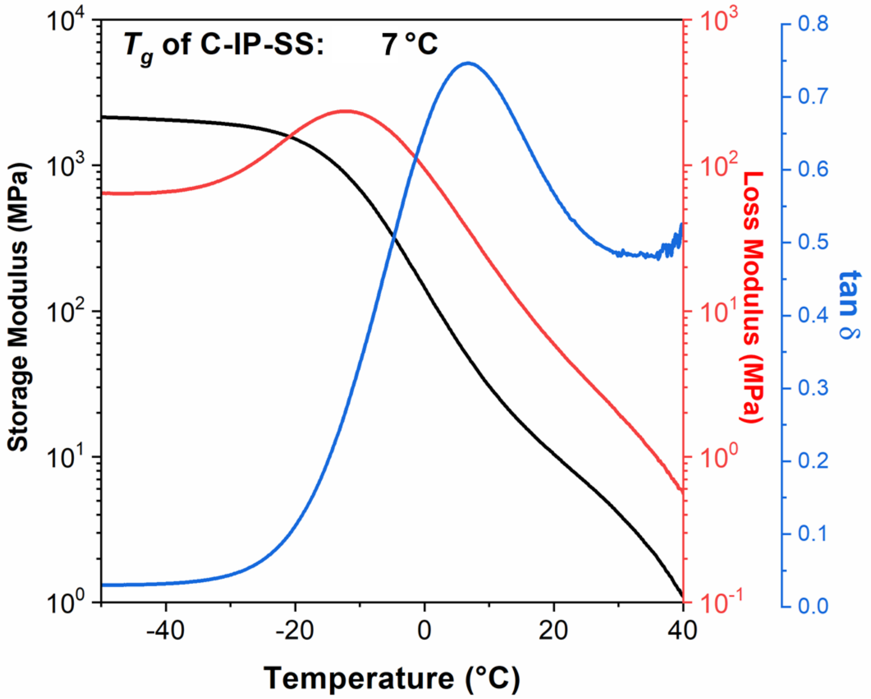

Supplementary Figure 21. DMA curves of C-IP-SS at 1 Hz over the temperature range of −80 to 80 ℃ in a liquid N_2 atmosphere.

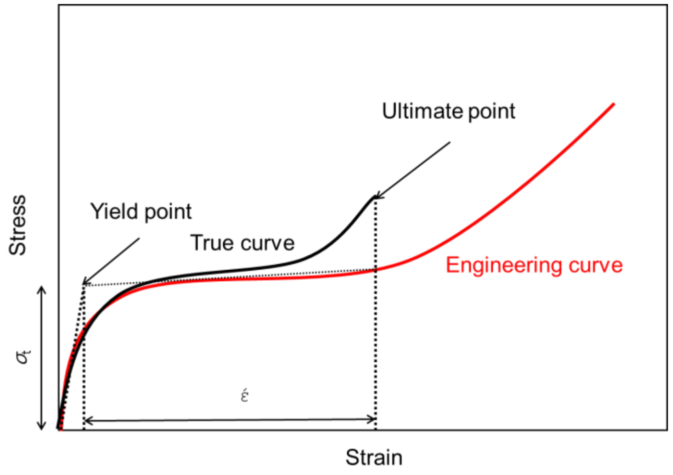

Supplementary Figure 22. Typical "engineering" and "true" stress–strain curves of C-IP-SS, and the transition stress and strain of C-IP-SS.

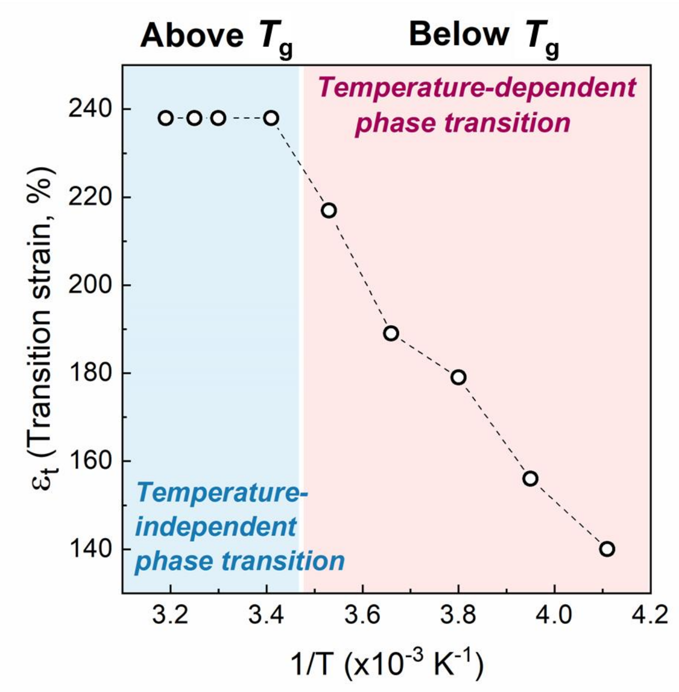

Supplementary Figure 23. Transition strain (έ) as a function of temperature (1/T) of C-IP-SS.

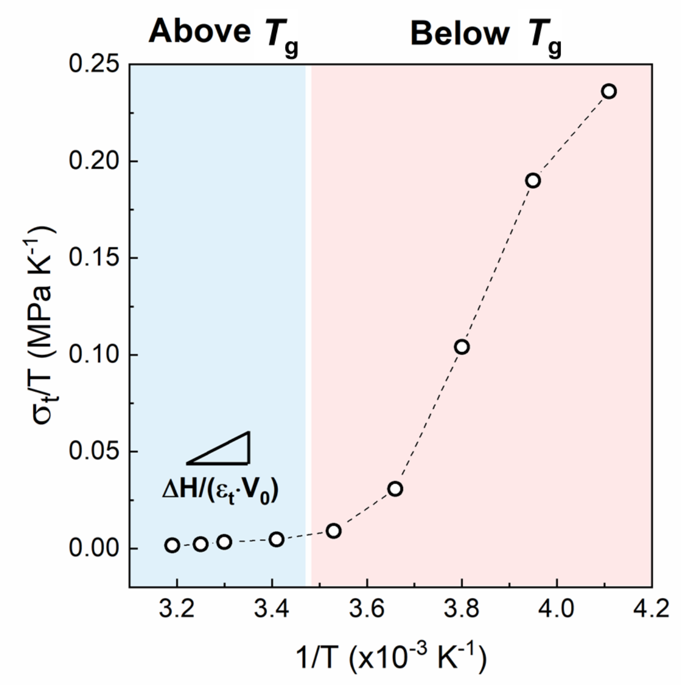

Supplementary Figure 24. Plot of (σ_t/T) as a function of temperature (1/T) of C-IP-SS.

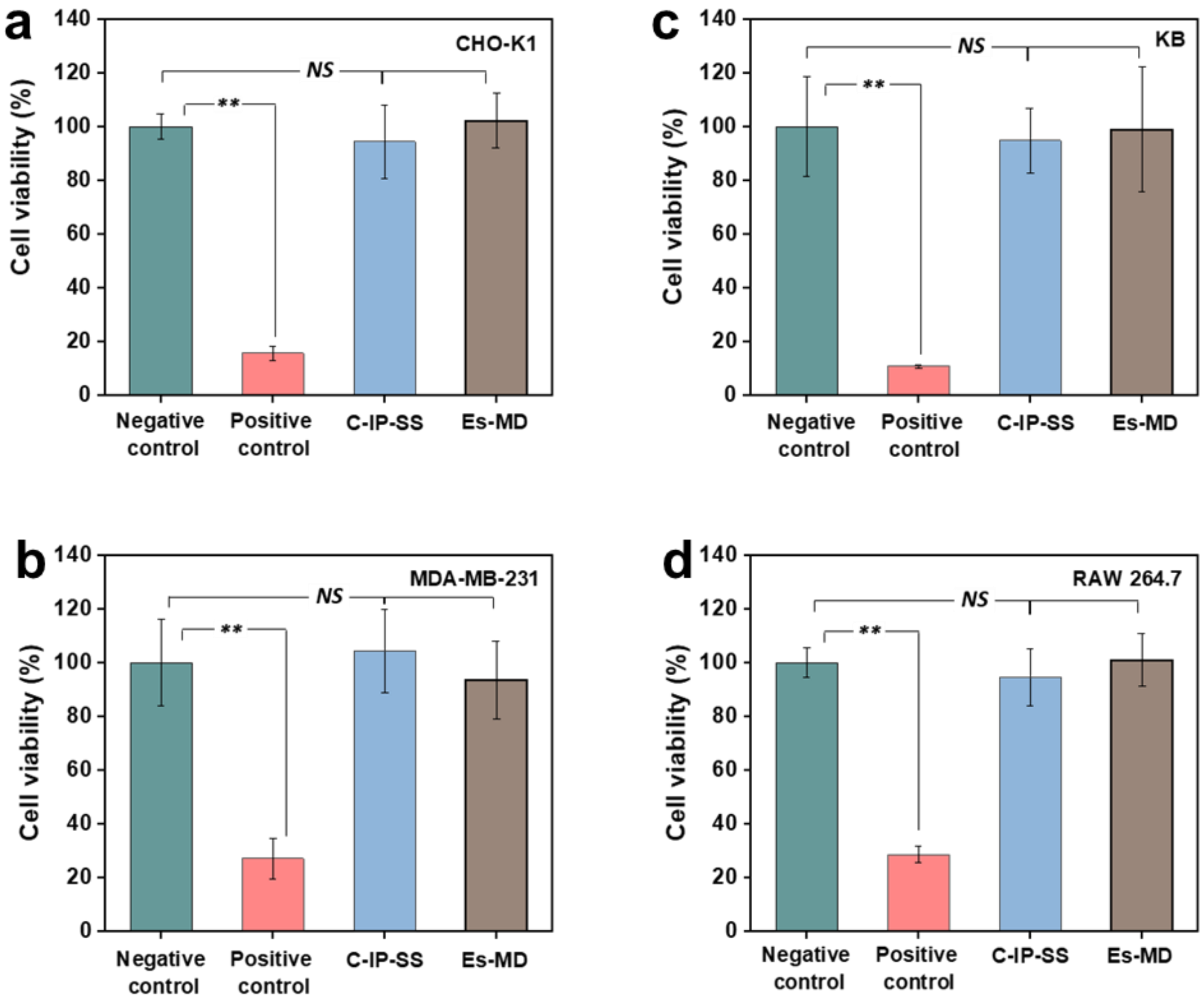

Supplementary Figure 25. *In vitro* cell viability tests of negative control (pristine cell culture medium), positive control (cell culture medium with 5% DMSO), C-IP-SS extracts, and Es-MD extracts using four different types of cells: (a) Chinese hamster ovary normal CHO-K1, (b) human breast carcinoma MDA-MB-231, (c) human epidermoid carcinoma KB, and (d) mouse macrophage normal RAW 264.7. The data of quintuplicate samples are expressed as mean ± the standard deviation. All data were evaluated using a Student's t-test at a not significance (NS) of $p > 0.05$ and a significance of $p < 0.01$ (**).

3. Supplementary Note

Supplementary Note 1: Calculation of an enthalpy of strain-induced phase conversion for C-IP-SS

The report by Miserez et al. evaluated an enthalpy change (ΔH) of phase transformation of reversibly deformable elastomers. Because this phenomenon is reminiscent of a shape-memory effect in a metallic alloy, it can be modelled by the Clausius-Clapeyron relation[21]. Our system was also applied to this relation because the mechano reversible change of internal structure of C-IP-SS thermodynamically resembles this biomaterial system.

Hence, based on the Gibbs free energy of a system, an applied force f can be express as a function of temperature and given by:

$$(\frac{\partial f}{\partial \mathrm{T}}) = \frac{f}{T} - \frac{\Delta H}{T \cdot \Delta L} \quad (1)$$

Or, being in a different form by considering the Clausius-Clapeyron relationship with force and length replacing pressure and volume:

$$(\frac{\partial (f/T)}{\partial (1/\mathrm{T})})_P = \frac{\Delta H}{\Delta L} \quad (2)$$

ΔL and ΔH respectively represent the changes in length and enthalpy during strain-induced crystallization; temperature T and pressure P.

In our system, f indicates the force at a yielding point for the conformational change during reversible crystallization. Hence, the equation (2) can be expressed with respect to stress and strain as:

$$(\frac{\partial (\sigma_t/T)}{\partial (1/\mathrm{T})})_P = \frac{\Delta H}{\varepsilon_t \cdot V_0} \quad (3)$$

σ_t and ε_t indicate the transition stress and strain, respectively, which are originally defined at stress-induced phase transition point defined in metal science. However, in polymer science, the σ_t and ε are respectively defined by the stress and strain at the point of strain-hardening where amorphous phase is transformed to crystal phase. Typically, strain-hardening occurs from the beginning of yield point to the end of ultimate strength point[22,23]. In the tensile stress-strain curves of C-IP-SS, it is difficult to define the both points because C-IP-SS has a too long strain. It can be clear by comparison between the true and engineering curves[21,24]. The engineering curves were corrected to the true stress-strain curves by the measured Poisson's ratios (Supplementary Fig. 22). As a result, the σ_t and ε for particular temperature were obtained in Supplementary Table S4.

Then, according to equation (3), we constructed the plot of (σ_t/T) versus the inverse of the temperature 1/T from our experimental results (Supplementary Figs. 23 and 24). The slope of the plot corresponds to the enthalpy change ΔH per unit volume V_0 when ε_t is temperature-independent. In the temperature range of above T_g where the reversible transition occurs, ε_t is constant and the plot of (σ_t/T) gives a linear curve.

Finally, by solving equation (3), we obtained the enthalpy of the strain-induced crystallization of C-IP-SS at above $T_g \approx 6.47$ cal cm^{-3} ($\Delta H/V_0$). Approximating the density of polyurethane (TPU) as 1.25 g cm^{-3}, the enthalpy can be converted to 5.17 g cm^{-3}.

Supplementary References

1. Wang, H., Yang, Y., Nishiura, M., Higaki, Y., Takahara, A. & Hou, Z. Synthesis of self-healing polymers by scandium-catalyzed copolymerization of ethylene and anisylpropylenes. *J. Am. Chem. Soc.* **141**, 3249-3257 (2019).
2. Zhang, L. et al. A highly efficient self-healing elastomer with unprecedented mechanical properties. *Adv. Mater.* **31**, 1901402 (2019).
3. Li, F. et al. Facile fabrication of self-healable and antibacterial soy protein-based films with high mechanical strength. *ACS Appl. Mater. Interfaces* **11**, 16107-16116 (2019).
4. Guo, H., Fang, X., Zhang, L. & Sun, J. Facile fabrication of room-temperature self-healing, mechanically robust, highly stretchable, and tough polymers using dual dynamic cross-linked polymer complexes. *ACS Appl. Mater. Interfaces* **11**, 33356-33363 (2019).
5. Fan, W. et al. Achieving fast self-healing and reprocessing of supertough water-dispersed" living" supramolecular polymers containing dynamic ditelluride bonds under visible light. *ACS Appl. Mater. Interfaces* **12**, 6383-6395 (2020).
6. Fan, W., Jin, Y., Shi, L., Du, W. & Zhou, R. Transparent, eco-friendly, super-tough “living” supramolecular polymers with fast room-temperature self-healability and reprocessability under visible light. *Polymer* **190**, 122199 (2020).
7. Yan, C., Yang, F., Wu, M., Yuan, Y., Chen, F. & Chen, Y. Phase-locked dynamic and mechanoresponsive bonds design toward robust and mechanoluminescent self-healing polyurethanes: A microscopic view of self-healing behaviors. *Macromolecules* **52**, 9376-9382 (2019).
8. Song, Y., Liu, Y., Qi, T. & Li, G. L. Towards dynamic but supertough healable polymers through biomimetic hierarchical hydrogen-bonding interactions. *Angew. Chem. Int. Ed.* **57**, 13838-13842 (2018).
9. Ha, Y.-m. et al. Robust and stretchable self-healing polyurethane based on polycarbonate diol with different soft-segment molecular weight for flexible devices. *Eur. Polym. J.* **118**, 36-44 (2019).
10. Lai, Y., Kuang, X., Zhu, P., Huang, M., Dong, X. & Wang, D. Colorless, transparent, robust, and fast scratch-self-healing elastomers via a phase-locked dynamic bonds design. *Adv. Mater.* **30**, 1802556 (2018).

11. Zou, C. & Chen, C. Polar-functionalized, crosslinkable, self-healing, and photoresponsive polyolefins. *Angew. Chem. Int. Ed.* **59**, 395-402 (2020).

12. Hu, J., Mo, R., Jiang, X., Sheng, X. & Zhang, X. Towards mechanical robust yet self-healing polyurethane elastomers via combination of dynamic main chain and dangling quadruple hydrogen bonds. *Polymer* **183**, 121912 (2019).

13. Liu, J. et al. An advanced elastomer with an unprecedented combination of excellent mechanical properties and high self-healing capability. *J. Mater. Chem. A* **5**, 25660-25671 (2017).

14. Liu, M. et al. A high stiffness and self-healable polyurethane based on disulfide bonds and hydrogen bonding. *Eur. Polym. J.*, **124,** 109475 (2020).

15. Peng, Y., Yang, Y., Wu, Q., Wang, S., Huang, G. & Wu, J. Strong and tough self-healing elastomers enabled by dual reversible networks formed by ionic interactions and dynamic covalent bonds. *Polymer* **157**, 172-179 (2018).

16. Fu, D. et al. A facile dynamic crosslinked healable poly (oxime-urethane) elastomer with high elastic recovery and recyclability. *J. Mater. Chem. A* **6**, 18154-18164 (2018).

17. Chen, Y., Tang, Z., Liu, Y., Wu, S. & Guo, B. Mechanically robust, self-healable, and reprocessable elastomers enabled by dynamic dual cross-links. *Macromolecules* **52**, 3805-3812 (2019).

18. Yanagisawa, Y., Nan, Y., Okuro, K. & Aida, T. Mechanically robust, readily repairable polymers via tailored noncovalent cross-linking. *Science* **359**, 72-76 (2018).

19. Urban, M. W., Davydovich, D., Yang, Y., Demir, T., Zhang, Y. & Casabianca, L. Key-and-lock commodity self-healing copolymers. *Science* **362**, 220-225 (2018).

20. Duan, N., Sun, Z., Ren, Y., Liu, Z., Liu, L. & Yan, F. Imidazolium-based ionic polyurethanes with high toughness, tunable healing efficiency and antibacterial activities. *Polym. Chem.* **11**, 867-875 (2020).

21. Miserez, A., Wasko, S. S., Carpenter, C. F. & Waite, J. H. Non-entropic and reversible long-range deformation of an encapsulating bioelastomer. *Nat. Mater.* **8**, 910-916 (2009).

22. Lim, H. & Hoag, S. W. Plasticizer effects on physical–mechanical properties of solvent cast soluplus® films. *AAPS PharmSciTech* **14**, 903-910 (2013).

23. Stachurski, Z. Strength and deformation of rigid polymers: The stress–strain curve in amorphous pmma. *Polymer* **44**, 6067-6076 (2003).

24. Roylance, D. *Stress-strain curves*. (Massachusetts Institute of Technology Press, Cambridge, 2001).
