# Supplementary material for: Mechano-responsive hydrogen-bonding array of thermoplastic polyurethane elastomer captures both strength and self-healing
Source: Nat Commun. 2021 Jan 27;12:621. doi: 10.1038/s41467-021-20931-z (PMC7841158; doi:10.1038/s41467-021-20931-z)
Supplement: Supplementary file 3 — Description of Additional Supplementary Files [file 41467_2021_20931_MOESM3_ESM.pdf]

Description of Additional Supplementary Files

File Name: Supplementary Movie 1
Description: Tensile testing of the recovered C-IP-SS after self-healing for 48 h at 35 °C.

File Name: Supplementary Movie 2
Description: A weight-lifting test of the recovered C-IP-SS using a 10-kg weight. The specimen with a thickness and a contact cut-area of 0.3 cm and 5 mm x 1 mm, respectively, was cut and self-healed for 48 h at 35 °C.

File Name: Supplementary Movie 3
Description: Manual drawing and twisting test of the recovered C-IP-SS at room temperature of 25 °C for 1 min. The specimen was cut and re-aligned with a contacted cut-area of 5 mm x 1 mm.

File Name: Supplementary Movie 4
Description: Macroscopic phase conversion and full recovery behaviour of C-IP-SS upon manual drawing.
