# Supplementary material for: Mechano-responsive hydrogen-bonding array of thermoplastic polyurethane elastomer captures both strength and self-healing
Source: Nat Commun. 2021 Jan 27;12:621. doi: 10.1038/s41467-021-20931-z (PMC7841158; doi:10.1038/s41467-021-20931-z)
Supplement: Supplementary file 8 — Reporting Summary [file 41467_2021_20931_MOESM8_ESM.pdf]

nature research

Corresponding author(s): Dongyeop Oh

Last updated by author(s): Dec 7, 2020

Reporting Summary

Nature Research wishes to improve the reproducibility of the work that we publish. This form provides structure for consistency and transparency in reporting. For further information on Nature Research policies, see our Editorial Policies and the Editorial Policy Checklist.

Statistics

For all statistical analyses, confirm that the following items are present in the figure legend, table legend, main text, or Methods section.

n/a	Confirmed	
☐	☒	The exact sample size (*n*) for each experimental group/condition, given as a discrete number and unit of measurement
☐	☒	A statement on whether measurements were taken from distinct samples or whether the same sample was measured repeatedly
☒	☐	The statistical test(s) used AND whether they are one- or two-sided<br>*Only common tests should be described solely by name; describe more complex techniques in the Methods section.*
☒	☐	A description of all covariates tested
☐	☒	A description of any assumptions or corrections, such as tests of normality and adjustment for multiple comparisons
☐	☒	A full description of the statistical parameters including central tendency (e.g. means) or other basic estimates (e.g. regression coefficient) AND variation (e.g. standard deviation) or associated estimates of uncertainty (e.g. confidence intervals)
☒	☐	For null hypothesis testing, the test statistic (e.g. *F*, *t*, *r*) with confidence intervals, effect sizes, degrees of freedom and *P* value noted<br>*Give P values as exact values whenever suitable.*
☒	☐	For Bayesian analysis, information on the choice of priors and Markov chain Monte Carlo settings
☒	☐	For hierarchical and complex designs, identification of the appropriate level for tests and full reporting of outcomes
☒	☐	Estimates of effect sizes (e.g. Cohen's *d*, Pearson's *r*), indicating how they were calculated

Our web collection on statistics for biologists contains articles on many of the points above.

Software and code

Policy information about availability of computer code

Data collection	Origin8.5 (GF3S5-3089-7904163), MS Office Pro 213 32bit (Y9NQC-FJ49B-DX87F-694JT-KTKKV)
Data analysis	Origin8.5 (GF3S5-3089-7904163), MS Office Pro 213 32bit (Y9NQC-FJ49B-DX87F-694JT-KTKKV)

For manuscripts utilizing custom algorithms or software that are central to the research but not yet described in published literature, software must be made available to editors and reviewers. We strongly encourage code deposition in a community repository (e.g. GitHub). See the Nature Research guidelines for submitting code & software for further information.

Data

Policy information about availability of data

All manuscripts must include a data availability statement. This statement should provide the following information, where applicable:
- Accession codes, unique identifiers, or web links for publicly available datasets
- A list of figures that have associated raw data
- A description of any restrictions on data availability

The source data that support the findings of this study are available (https://doi.org/ 10.6084/m9.figshare.12936989). We provide the source data underlying Figs. 1b, 1c, 3a, 4a, 4b, 5a, 5b, 5c, 5e, and 6b–d, and Supplementary Figs. 2–6, 9–11, 13, 14, 16, 18, 21, and 23–25.

Field-specific reporting

Life sciences study design

All studies must disclose on these points even when the disclosure is negative.

Sample size	1. in vivo experiments<br>n = 4 (the number of SD rat), SD rat weight 250-300 g<br>ISO 10993-6:2007 Tests for local effects after implantation<br><br>2. in vitro cell line experiments. a cytotoxic effect (ISO 10993-5).<br>n = 5, (the number of wells for growing cell lines)
Data exclusions	No data exclusion was conducted.
Replication	All biological experiments were conducted once. They were not replicate.
Randomization	1. in vivo experiments<br>All rats were chosen randomly. The weight of all rats were not different from that of each in t-test (p-value > 0.05) within the whole experimental procedures.<br><br>2. in vitro cell line experiments<br>The all experiments for the negative control group and the experimental group were conducted using the same homogeneously cell containing medium.
Blinding	1. All in vivo experiments were conducted by a a (public) contract clinical research organization, Daegu Gyeongbuk medical innovation foundation (DGMIF) (http://www.dgmif.re.kr/eng/index.do), following ISO 10993-6:2007 Tests for local effects after implantation.<br>The data processing and analyses were conducted by a professional contract pathologist.<br>The professional contract pathologist have no information for samples and only know the sample codes.<br><br>2.<br>No blinding tests were conducted in the vitro experiments.

Reporting for specific materials, systems and methods

We require information from authors about some types of materials, experimental systems and methods used in many studies. Here, indicate whether each material, system or method listed is relevant to your study. If you are not sure if a list item applies to your research, read the appropriate section before selecting a response.

Materials & experimental systems

n/a	Involved in the study	
☒	☐	Antibodies
☐	☒	Eukaryotic cell lines
☒	☐	Palaeontology and archaeology
☐	☒	Animals and other organisms
☒	☐	Human research participants
☒	☐	Clinical data
☒	☐	Dual use research of concern

Methods

n/a	Involved in the study	
☒	☐	ChIP-seq
☒	☐	Flow cytometry
☒	☐	MRI-based neuroimaging

Eukaryotic cell lines

Policy information about cell lines

Cell line source(s)	Human breast carcinoma MDA-MB-231 cells, human epidermoid carcinoma KB cells, Chinese hamster ovary normal CHO-K1 cells, and mouse macrophage normal RAW 264.7 cells
Authentication	We bought the authenticated cells from Korea Cell Line Bank
Mycoplasma contamination	As Korea Cell Line Bank provides, the cell lines were not contaminated by Mycoplasma.
Commonly misidentified lines<br>(See ICLAC register)	We don't use any commonly misidentified lines.

Animals and other organisms

Policy information about studies involving animals; ARRIVE guidelines recommended for reporting animal research

Laboratory animals	Sprague Dawley (SD) Rat Model, Male, 9 weeks of age,
Wild animals	n/a The study did not involve wild animals
Field-collected samples	n/a The study did not involve field-collected samples
Ethics oversight	The in vivo biocompatibility test was conducted by the Daegu Gyeongbuk Medical Innovation Foundation (DGMIF), a contract clinical research organization, following the ISO 10993-6 Annex A standard63, and ethically approved by the Institutional Animal Care and Use Committee (Korea) (IACUC; approval code DGMIF-19042402-00).

Note that full information on the approval of the study protocol must also be provided in the manuscript.
